# An Approach to the Prototyping of an Optimized Limited Stroke Actuator to Drive a Low Pressure Exhaust Gas Recirculation Valve

**DOI:** 10.3390/s16050735

**Published:** 2016-05-20

**Authors:** Christophe Gutfrind, Laurent Dufour, Vincent Liebart, Jean-Claude Vannier, Pierre Vidal

**Affiliations:** 1 EFI Automotive, 77 allée des grandes Combes, 01708 Miribel, France; laurent.dufour@efiautomotive.com (L.D.); vincent.liebart@efiautomotive.com (V.L.); 2 Centrale Supelec, 1 rue Joliot-Curie, 91192 Gif sur Yvette, France; jean-claude.vannier@centralesupelec.fr (J.-C.V.); pierre.vidal@centralesupelec.fr (P.V.)

**Keywords:** axial flux machine, limited stroke, optimization, finite element method, prototyping, automotive application

## Abstract

The purpose of this article is to describe the design of a limited stroke actuator and the corresponding prototype to drive a Low Pressure (LP) Exhaust Gas Recirculation (EGR) valve for use in Internal Combustion Engines (ICEs). The direct drive actuator topology is an axial flux machine with two air gaps in order to minimize the rotor inertia and a bipolar surface-mounted permanent magnet in order to respect an 80° angular stroke. Firstly, the actuator will be described and optimized under constraints of a 150 ms time response, a 0.363 N·m minimal torque on an angular range from 0° to 80° and prototyping constraints. Secondly, the finite element method (FEM) using the FLUX-3D^®^ software (CEDRAT, Meylan, France) will be used to check the actuator performances with consideration of the nonlinear effect of the iron material. Thirdly, a prototype will be made and characterized to compare its measurement results with the analytical model and the FEM model results. With these electromechanical behavior measurements, a numerical model is created with Simulink^®^ in order to simulate an EGR system with this direct drive actuator under all operating conditions. Last but not least, the energy consumption of this machine will be estimated to evaluate the efficiency of the proposed EGR electromechanical system.

## 1. Introduction

### 1.1. Automotive Context

**Figure 1:**
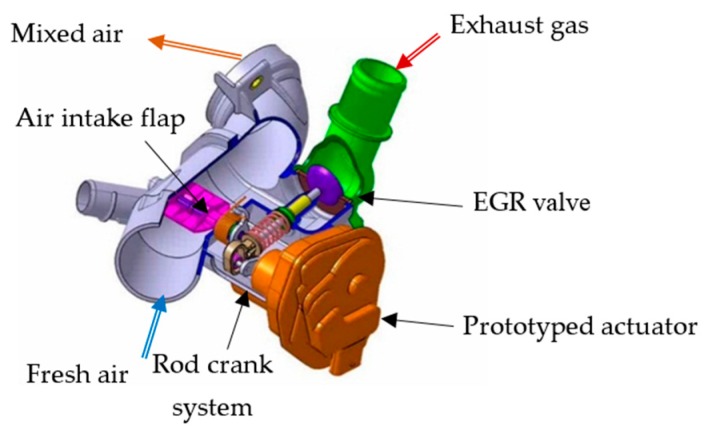
Air flow and EGR system of an internal combustion engine with the actuator prototype.

In order to reduce greenhouse gases and the polluting emissions of new vehicles, new internal combustion engine concepts need a new regulation management of air flow as described in [1,2]. As shown in [1] and Figure 1, Mann and Hummel proposed a dual air loop EGR system using an EGR Low Pressure (LP) valve and air flow throttle. This system is more adapted to plastic use as opposed to the topology using an exhaust throttle as presented in [2]. The use of an intake valve also leads to lesser stress on the DC motor and electronic components.

As we can see from the drawing in Figure 1, the actuator moves this mechanism in two steps through a rod-crank system. In the first step, the actuator moves the EGR valve and opens the EGR circuit loop in order to mix exhaust gas with fresh air. Then, when the EGR valve is wide open, as it can be necessary to improve the EGR rate, the actuator closes the fresh air circuit with the air intake flap.

**Figure 2:**
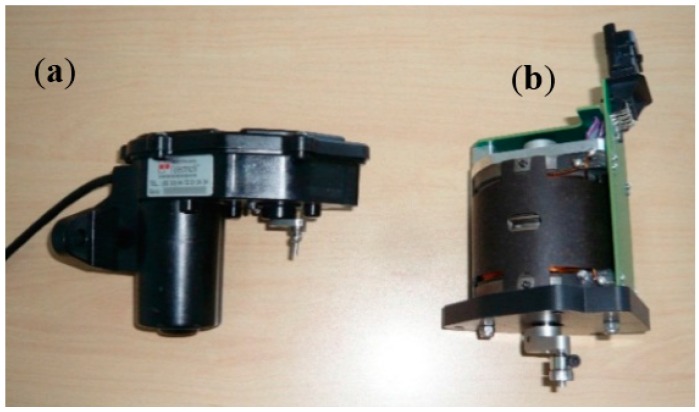
Indirect and direct drive actuator prototypes: (**a**), the indirect drive actuator, (**b**) the direct drive actuator. As presented in [7], both actuators are optimized with the same requirements in order to compare their energy consumptions.

To drive this EGR mechanical system, a state of the art electromechanical actuator, that manages the air flow, as described in [3,4,5], has been identified and several electrical DC machine structures analyzed. There are two main electric topologies which produce rotary movements: the first one is an indirect drive [5], composed of a brushed DC machine as in [6] and a reduction gear set, the second one is a direct drive machine such as a torque motor defined by a single phase BLDC machine. The two topologies are prototyped and presented in Figure 2.

For the application and this paper, the direct drive actuator has been chosen for its reliability as well as its time response qualities in order to avoid the non-linear behaviour of the gear set of an indirect drive actuator. The 80° angular stroke requirement implies that the actuator is defined by a bipolar permanent magnet in order to produce a constant and high torque for a given value of current on a 180° theoretical stroke (more or less 120° in reality). This actuator is designed in two steps.

### 1.2. Choice of Actuator Topology

First, in order to choose an electrical machine concept, references [8,9,10] describe some Limited Angle Torque Motor (LATM) topologies with a toroidally wound armature. This topology has its pros and cons. In many ways, from the torque per unit volume and manufacture point of view, the concentrated winding is preferred to respect some requirements constraints. A comparative study of five magnetic structures is described in [11] with two radial flux machines with an internal or external rotor and three axial flux machines topologies with one or two air gaps. Each topology is composed of concentrated windings and a ferromagnetic core on the stator, and then a surface mounted permanent magnet on the rotor. A ferromagnetic part on the rotor might be used in some cases. The comparative study shows a simple analytical model in which the sizing parameters of the actuator geometry and the electromagnetic characteristics are defined. Then an optimization objective is used to calculate the optimal values of these parameters in order to minimize the actuator volume according to the physical and merely dynamic constraints.

In this case, the magnetic concept results show that radial flux machines are better than axial flux machines for their performance in restricted volumes. Nevertheless, axial flux machines are more interesting for their flat form magnets and the concentrated windings that are simpler to produce. The final result of this comparative study on actuating concepts shows that an axial flux machine with two air gaps with a central rotor without ferromagnetic part is more attractive for its flat form magnet and its low rotor inertia.

### 1.3. Axial Flux Machine Design Approach

**Figure 3:**
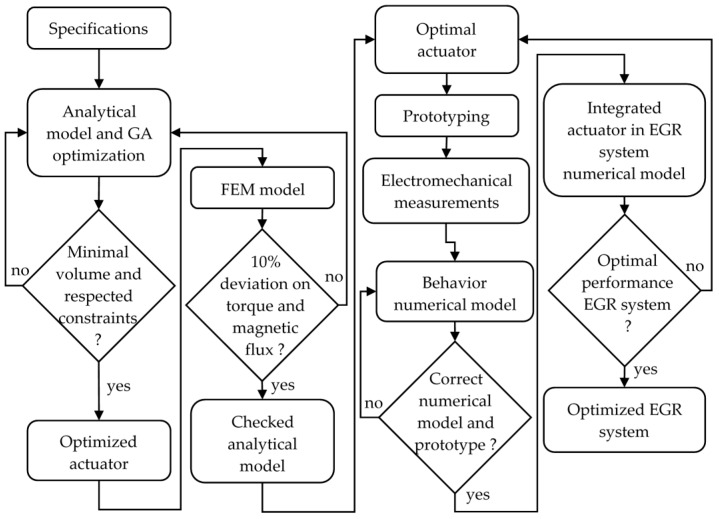
The flowchart procedure to design the prototype direct drive actuator.

In a second part, an optimization to minimize the actuator volume and to consider the others constraints such as the prototyping and manufacturer constraints is demonstrated. The results are checked with the FEM with FLUX-3D in non-linear mode. Then the prototype and its characterization can be realized in order to simulate the EGR mechanical behavior under all conditions. The design flowchart to define the optimal actuator to drive an EGR mechanical system is summarized in Figure 3.

## 2. Analytical Model

**Figure 4:**
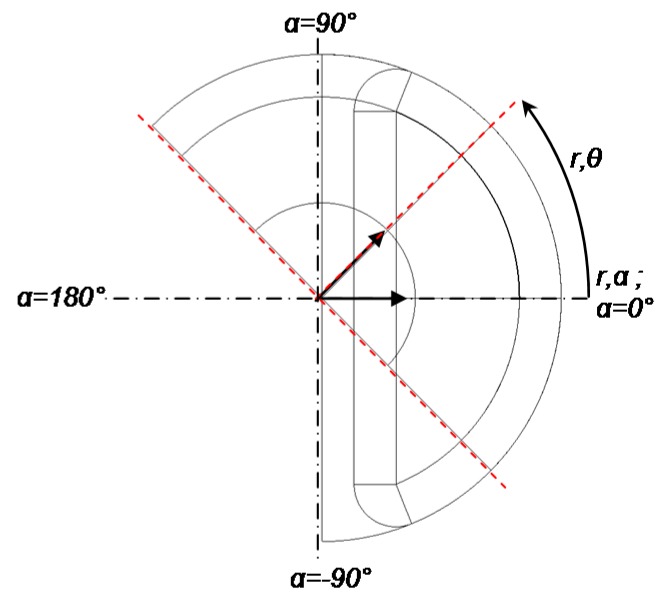
Rotor and stator cylindrical references.

To write the flux and torque expressions, the air gap magnetic flux density must be defined. A cylindrical reference (r,α) is fixed to the stator in order to visualize the magnetic flux density. A moving cylindrical reference (r,θ) is fixed to the rotor to visualize the actuator torque. θ is a rotational angle of the rotor with respect to α = 0°. Figure 4 presents the rotor and stator cylindrical references.

**Figure 5:**
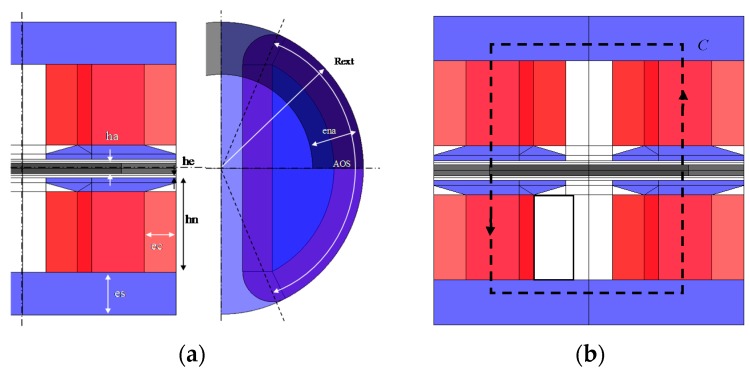
Direct drive actuator parameters (**a**) and the flux line contour C in axial flux machine (**b**).

The actuator is a bipolar permanent magnet composed of four coiled concentrated windings and two magnets in the rotor. Figure 5 shows the axial flux actuator topology.

### 2.1. Air Gap Magnetic Flux Density Calculation

**Figure 6:**
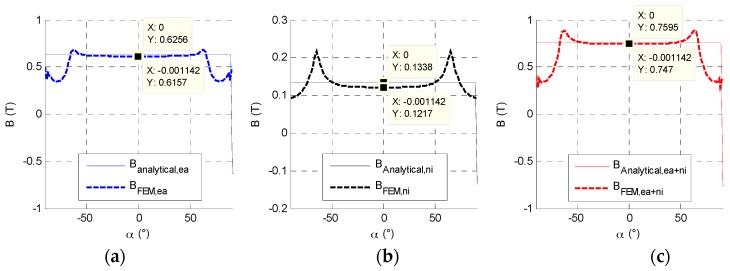
The distributed induction flux density in the air gap: (**a**) magnet induction flux density *B_ea_*; (**b**) the magnetomotrice force flux density *B_ni_*; (**c**) sum of both *B_e_* on a complete pole pitch.

As presented in [4], the analytical model calculates the actuator torque in accordance with the magnetic flux produced by magnets and the magnetomotive force *fmm*. The relation of the magnetomotive force depends on *n*, which is the number of turns in a slot pair and *I*, the current in each coil for *k_nI_* coils. Ampere’s law on *C* path is applied for each actuator, flux crosses *k_e_* times an air gap thickness *e* and *k_a_* times magnet thickness *e_a_*:

(1) $$k_{a}\cdot H_{a}\cdot e_{a}+k_{e}\cdot H_{e}\cdot e=fmm=k_{nI}\cdot n.\cdot I$$

where *H_a_* is the magnet field and *H_e_* the air gap field. Then, with the magnetic field in air gap law, magnet field law, and conservative flux law, the magnetic flux density in the air gap *B_e_* can be defined by the sum of the magnets’ flux density part and the *fmm* part such as:

(2) $$\begin{array}{l} B_{e}(\theta ,\alpha )=B_{ea}\cdot \beta (\theta )+B_{ni}\cdot \delta (\theta ,\alpha )\\ \text{With }B_{ea}=\frac{k_{a}\cdot B_{r}\cdot e_{a}}{k_{a}\cdot k_{ea}\cdot e_{a}+k_{e}\cdot \mu_{a}\cdot e}\;and\;B_{nI}=\frac{k_{nI}\cdot \mu_{0}\cdot n\cdot I}{\left(k_{e}\cdot e+k_{a}\cdot k_{ea}\cdot \frac{e_{a}}{\mu_{a}}\right)} \end{array}$$

where *µ_0_* is the vacuum permeability, *µ_r_* the magnet relative permeability and *B_r_* the remanent flux density of magnet and *B_a_* the magnetic flux density in magnet. Functions β and δ equal −1 or 1 according to the observation position in the air gap as shown in Figure 6. The magnets’ flux density, *B_ea_*, depends on the rotor position according to *θ*. The stator current reaction, *B_ni_*, is fixed whatever the rotor position. The resulting flux density in the air gap, *B_e_*, is the sum of both.

### 2.2. Excitation Flux Expression and Saturation Constraints

The magnet flux depends on *θ* the rotor position with regard to stator. The excitation flux in air gap is defined by:

(3) $$\Phi_{e}\left(\theta \right)=\int_{-\pi /2}^{\pi /2}S_{r}\cdot B_{e}\;(\theta ,\alpha )\;d\theta =S_{r}\cdot B_{e}\cdot \left(\pi -2\theta \right)$$

**Figure 7:**
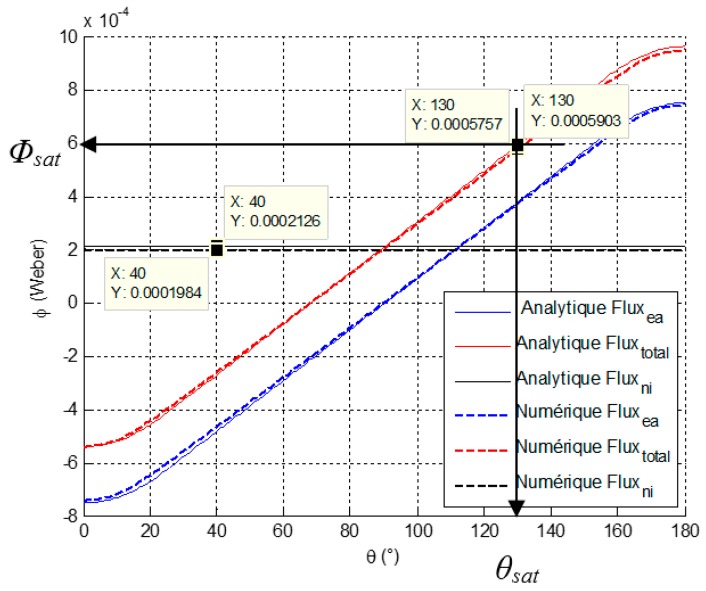
Magnetic flux evolution observed by a coiled polar tooth.

The magnets flux *Φ_ea_*, depends on the rotor position, the curve is variable according to θ. The stator current reaction, *Φ_ni_*, (with *Φ_leakage_* integrated) is fixed whatever the rotor position. The resulting flux *Φ_e_* is the sum of both. Figure 7 presents each magnetic flux part.

The magnetic flux is used to define the actuator geometry according to the saturation induction level. The maximum magnetic flux densities in the rotor and stator are calculated with the *Φ_sat_* maximal flux that crosses the rotor and stator section at the last rotor position *θ_sat_* according to the end of stroke. For example at the *θ_sat_* rotor position equal to 130°, the magnetic circuit is designed with the *Φ_sat_* magnetic flux value at this point. Saturation flux density is calculated in the magnetic circuit such as:

(4) $$B_{i}=\frac{{\Phi_{sat}|}_{\theta_{sat}}}{S_{i}}<B_{sat}$$

### 2.3. Torque Definition

The torque value is defined by the variation of the magnetic flux. In magnetic coenergy derivative, the part of the flux varies according to the rotor position *θ* from between 0° and 180° such as:

(5) $$T_{m}={\left(\frac{\partial W_{m}}{\partial \theta }\right)}_{I=cst}=n\cdot I\cdot \frac{\partial \Phi_{e}}{\partial \theta }$$

**Figure 8:**
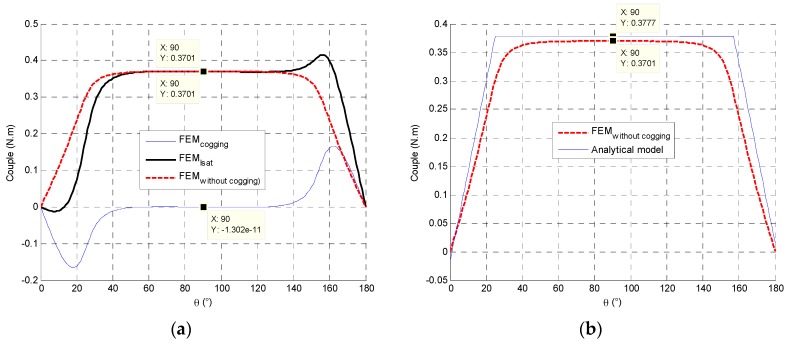
(**a**) The FEM torque evolution between cogging torque and torque at fixed current; (**b**) The torque comparison in a linear behavior between the analytical model and FEM model.

The cogging torque is not modelled in the analytical model. The FEM resolution is linear and the obtained torque includes the cogging effect. In order to compare analytical and FEM torques, the cogging torque values are subtracted from the torque FEM result at saturation current. Figure 8 presents the torque evolution according to the rotor position and the influence of a polar tooth’s opening angle.

Then, the linear constant torque between torque computation and the current in the coil can be obtained:

(6) $$K_{t}=\frac{T}{I}$$

### 2.4. Magnets Thickness Constraints

Permanent magnets are made of rare earth alloys because of their high energy characteristics. The magnet model is simplified for the analytical study. From Equation (2), the magnet minimal thickness should verify the following condition to avoid demagnetization:

(7) $$e_{a}>\frac{k_{nI}\cdot n\cdot I}{k_{a}\cdot H_{cB}}$$

### 2.5. Electrical Resistance and Inductance Coils

The geometrical definition implies an *l* wire length. Its length depends of quantity of coil turns *n* that it is possible to place in the slot section in accordance with the enameled copper wire section *S_cu_*. The electrical resistance of the actuator, composed of four coils, is written as:

(8) $$R=\rho_{\left(\Omega .m\right)}\cdot \frac{l}{S_{cu}}$$

The electrical resistivity at 130 °C is 24.3 × 10^−8^ Ω·m. The inductance coils expression is written in accordance with the magnetic flux *Φ* and the current *I*:

(9) $$L=\frac{\Phi }{I}$$

### 2.6. Total Inertia at the Actuator Shaft End

To move the EGR system with a 150 ms time response on 80° stroke, the actuator develops a torque according to the sum of all inertia. The total inertia of the system is composed by the magnet inertia and a drive shaft inertia such as *J_m_* and then the EGR system inertia such as *J_load_* such as:

(10) $$J_{tot}=J_{magnet}+J_{shaft}+J_{load}$$

Then, according to a uniform acceleration and deceleration, the actuator torque should be higher than the sum of inertia and load *T_load_* such as:

(11) $$T_{m}>\left(J_{tot}\cdot Acc+T_{load}\right)$$

### 2.7. Electromechanical Behavior

**Figure 9:**
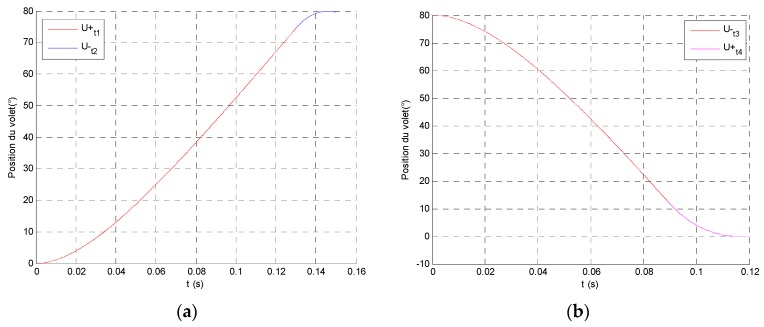
The uniform accelerating and decelerating trajectories; (**a**) forward and (**b**) backward travels.

At this stage, the optimization doesn’t consider a closed loop control. Nevertheless, in order to respect the required response time like a closed loop control system as presented in [12], a position cycle is described with a 150 ms flap position response time including acceleration and deceleration for the forward and backward travel as shown in Figure 9. The optimization method has to calculate the switching time in compliance with the flap position specification on a forward and backward travel from 0° to 80°.

Electrical and mechanical balances are expressed as:

(12) $$\left\{ \begin{array}{l} J_{tot}\cdot {\dot{\Omega }}_{m}=K_{t}\cdot I-C_{ch}\\ U=R\cdot I+L\cdot \dot{I}+K_{e}\cdot \Omega_{m} \end{array}\right.$$

To calculate the rotor position *versus* supply voltage time, the electrical balance and mechanical equations must be solved. Inductance values cannot be ignored and this involves a state-space model to compute the acceleration, speed and then the position of actuator. The electromechanical balance is written by this state-space matrix:

(13) $$\left(\begin{array}{c} {\dot{\Omega }}_{m}\\ \Omega_{m}\\ \dot{I} \end{array}\right)=\left(\begin{array}{ccc} 0 & 0 & \frac{K_{t}}{J_{tot}}\\ 1 & 0 & 0\\ -\frac{K_{e}}{L} & 0 & -\frac{R}{L} \end{array}\right)\cdot \left(\begin{array}{c} \Omega_{m}\\ \theta_{m}\\ I \end{array}\right)+\left(\begin{array}{c} -\frac{C_{ch}}{J_{tot}\cdot U}\\ 0\\ \frac{1}{L} \end{array}\right)\cdot U$$

## 3. Optimization and Results

The state of the art of the Genetic Algorithm (GA) is described in [13]. The GA is a one of the most popular algorithms to optimize electrical devices and it has been used to optimize the model parameters of the analytical model.

### 3.1. Algorithm Optimization

A numerical software such as MATLAB® is used for the optimization. The GA is coded in real time with it. The GA is a sequence of a selection, mutation and crossover of individuals from a population as described in [13]. An individual is a parameter vector composed by genes. A gene is a variable parameter of the actuator who takes an integer value between a minimal and a maximal limit. Elitism is used to conserve the best individuals of a generation. This strategy copies the best individual from generation n − 1 into generation *n*. GA converges to the global best individual who defines the optimized actuator. The optimization objective is to minimize the volume. The population is composed of 200 individuals. The crossover coefficient is equal to 70% and the mutation to 0.1%. 50% of best individuals, who respect the constraints, are conserved from generation n − 1 into generation *n* and the 50% part of the population are created according to the crossover and the mutation. If the minimal volume doesn’t vary anymore after 200 generations, GA stops the optimization. We then consider that the objective has been reached.

### 3.2. Functioning Conditions and Constraints

The axial flux machine is optimized in the worst operating case of the EGR system. The functioning condition are:

- a 130 °C temperature,
- a 9 V supply voltage,
- a 363 mN·m minimal torque at the end of stroke,
- a 150 ms time response for a 80° angular stroke.

In order to define the number of coils turns, a continuous evolution of enameled copper wire diameter is defined between 0.1 and 2.5 mm. Besides, the magnet material is defined with a 1 T magnet remanent flux density *B_r_* at 130 °C with a relative permeability *µ_a_* equal at 1.03. A 0.8 mm air gap thickness *e* is chosen. The constraints are the following:

- the stator and rotor flux density should be lower than 1.57 T at the end of stroke, because of the saturation magnetic flux *Φ_sat_* with a 4500 maximal relative permeability, as defined in [14],
- the magnet thickness (Equation (6)) should be higher than the demagnetization magnet thickness,
- the actuator torque should be higher than the sum inertial of torque and required torque on a 92° minimum stroke range (to include edge effect),
- the electromechanical time constant is three times lower than the response time,
- the current density is limited at 5 A/mm^2^ in the slot section,
- the maximal current is 10 A.

To respect dynamic needs and to avoid too restrictive a calculation, 5% tolerance on the output shaft position at the end of stroke is used during the calculation. This tolerance affects the forward and backward acceleration times and consequently, the optimization can accept a large of number of good candidates who respect electromechanical constraints.

### 3.3. Optimization Results

At the optimization start point, the analytical model computes a 238 cm^3^ actuator volume. After 3233 generations and a 67,326 s computation time, at the end of optimization, the optimal actuator volume is 198 cm^3^ with the respected constraints. Table 1 shows the optimized variable parameter values.

The optimized direct drive actuator is characterized with a 90 mN·m/A constant torque, a 1.12 ohm electrical resistance and a 23.7 mH electrical inductance at 25 °C, and then, a 0.8 × 10^−5^ kg·m^2^ rotor inertia.

### 3.4. FEM Checking in Linear and Saturated Behavior

The analytical model actuator is defined with a linear magnetic material model without B-H saturation curve. Table 2 shows the difference between the analytical and FEM linear results for a 5.72 A saturation current. The linear behavior, analytical results and FEM results are similar.

**Figure 10:**
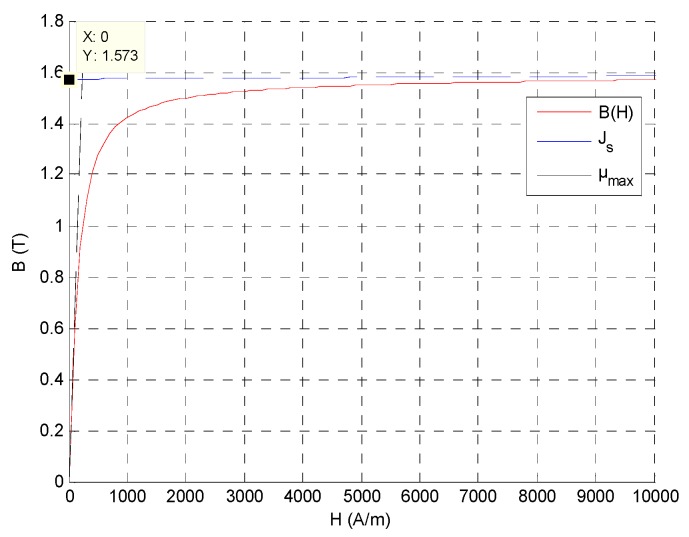
The B-H saturation curve for a 1.57 T saturation induction and a 4500 relative permeability.

Moreover, if the actuator is a good design, at the saturation current and for the 130° rotor position, the actuator core reaches a 1.57 flux density. At the moment, FLUX-3D can’t compute a B-H hysteresis loop of magnetic materials. A B-H saturation curve is defined by an Arctan function such as used in FLUX-3D, as defined in [15] and presented in Figure 10 below.

**Figure 11:**
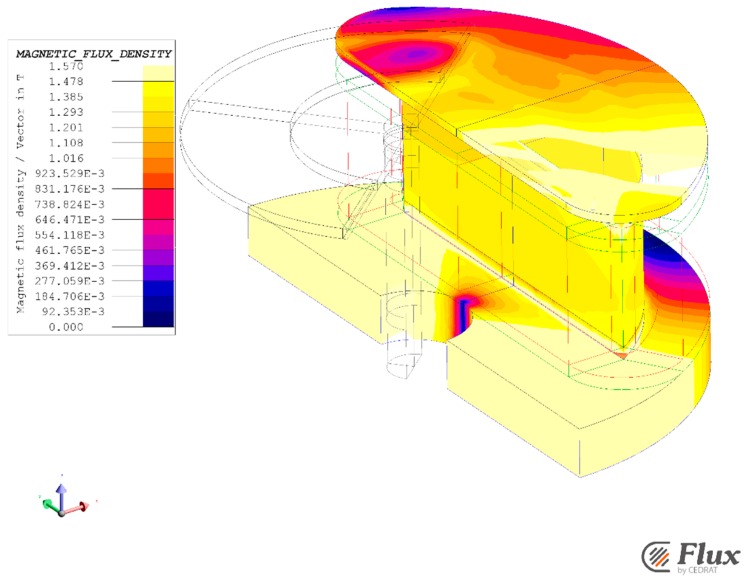
Magnetic flux density in core at the 130° rotor position and the 5.72 A current.

As shown in Figure 11, all in all, the stator flux density level shows at the 130° rotor position that the actuator geometry seems to respect the saturation constraints at less than 1.57 T in the core.

**Figure 12:**
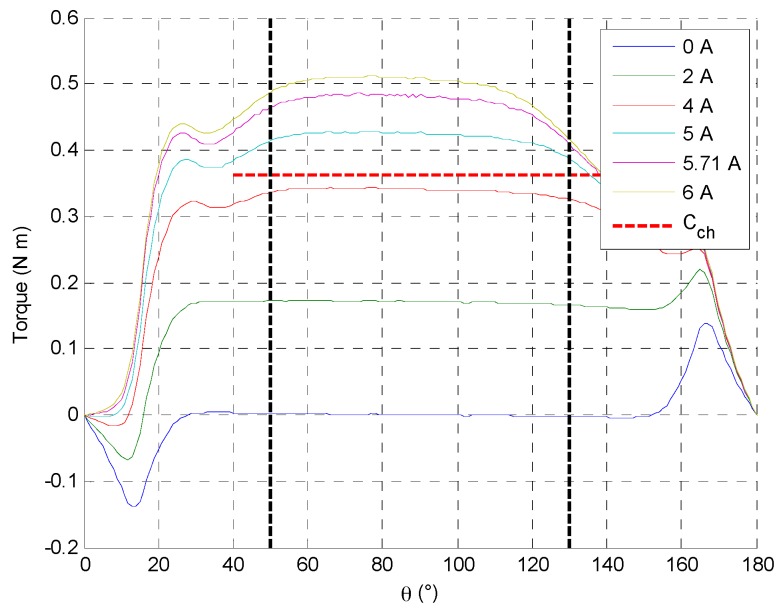
Torque actuator evolution at different positive current and rotor position.

However, the analytical model is a mean model, so the located saturation can’t be estimated. In the linear behavior, the flux density in the first part of the teeth is higher than the 1.57 T and this point can be overlook since the 80° angular range of the actuator is between from 50° to the 130° rotor position such as presented in Figure 12.

The comparison between analytical model and FEM model are correct as presented in Table 3. The prototype can be realized and characterized.

## 4. Actuators Characterization to Build an Electromechanical Behavior Model

To drive the prototyped actuator, a power electronics and a control are defined. The EGR system needs a reversibility control in forward and backward travel. The power electronics is defined by a H-bridge with a low ohmic resistance (0.2 Ohm including wiring). Then, in order to control the shaft position, a control system is defined with a Hall Effect position sensor, developed by EFI Automotive [16], integrated in output shaft end of the direct drive actuator and a microcontroller with the control law implemented.

### 4.1. Mechanical and Electric Behaviors Measurements

The electrical resistance and magnet remanent flux density display a linear behavior according to the temperature. However, the ferromagnetic characteristics of the core give some nonlinear effects on torque and inductance values in accordance with the core saturation flux density.

#### 4.1.1. Non-Linear Torque

**Figure 13:**
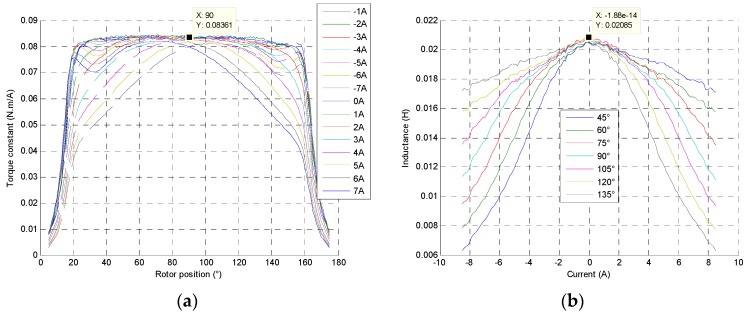
(**a**) Constant torque evolution at each rotor position and −7 A to 7 A current of the direct drive actuator at 25 °C; (**b**) Inductance coil at each rotor position and current.

At each rotor position and current, the torques developed by the actuator are measured on a test bench and torque constants can be obtained as shown in Figure 13. At the beginning of a stroke, between 20° and 40°, the actuator torque decreases because of the concentrated windings saturation, and then, at the end of stroke, the core saturation implies naturally the decreasing torque. For positive current, the results give a decreasing torque as the rotor position and current increase. Then, the actuator constant torques can be computed at different currents and rotor positions as shown in Figure 13a at 25 °C.

#### 4.1.2. Non-Linear Inductance

As shown in Figure 13b, the same behavior can be observed in the inductance evolution. Its value decreases as the rotor position and the current increase. The inductance coil values varies according to the core flux density and material saturation level.

#### 4.1.3. Magnetic Hysteresis Influence

**Figure 14:**
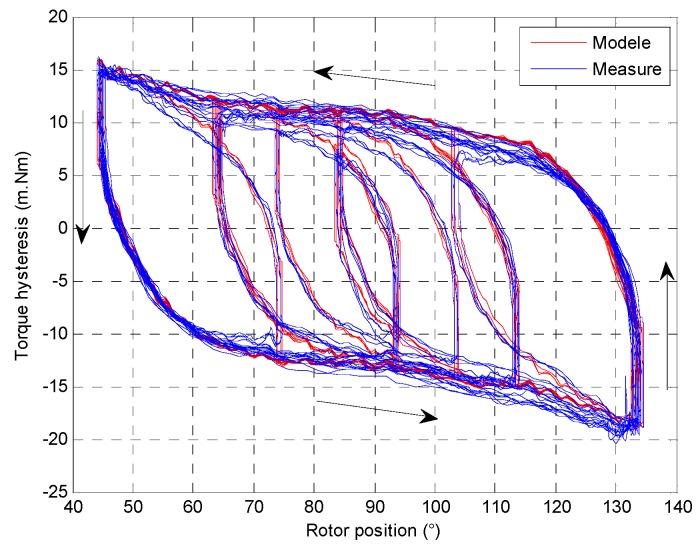
Hysteresis torque according to rotor position.

The core is defined by a ferromagnetic material and has a magnetic hysteresis around 100 A/m according to the material manufacturer. A magnetic hysteresis implies a hysteresis torque depending on rotor position such as presented in Figure 14. If the rotor position has to change, the current must be higher than necessary to move the rotor. The B-H hysteresis curve looks like as a memory effect to the electromechanical behavior. The hysteresis torque is variable according the rotor position and the previous position.

To define the electromechanical behavior with non-linearity effects, the actuator is characterized with the torque and current consumption measurements. Then, with Simulink, the electromechanical model use two interpolates tables with inductance, constant torque values and the hysteresis curve.

### 4.2. Controllers

The control law definition is modelized with a linear state space model. In this case, the inductance value involves an electrical time constant (18 ms) of the same order of magnitude as electromechanical time constant (12 ms). The system is considered as a second order system. The direct drive transfer function is defined with nominal parameters:

(14) $$\begin{array}{l} H\left(s\right)=\frac{H\left(0\right)}{1+\tau_{1}s+{\tau_{2}}^{2}s^{2}}=\frac{H\left(0\right){\omega_{n}}^{2}}{s^{2}+2\omega_{n}\xi s+{\omega_{n}}^{2}}\\ \text{with }H\left(0\right)=\frac{1}{K_{t}};\;\tau_{1}=\frac{JR}{K_{t}^{2}};\;{\tau_{2}}^{2}=\frac{JL}{K_{t}^{2}}\text{ and }\xi =\frac{\tau_{1}}{2\tau_{2}};\;\omega_{n}=\frac{1}{\tau_{2}} \end{array}$$

The system angular frequency *ω_n_* value is 71 rad/s. The damping factor *ξ* value is 0.43, lower than 0.7, highlighting an oscillating system. For this DC machine, a cascade control is defined with two control loops. The primary loop is composed with P controller (with *K_i_* proportional gain) and current measurement. The second loop, devoted to the position control, is defined with PID controller (with *K*_θ_ proportional gain, *T*_θ_ Integral gain, *D*_θ_ Derivative gain) and position measurement.

**Figure 15:**
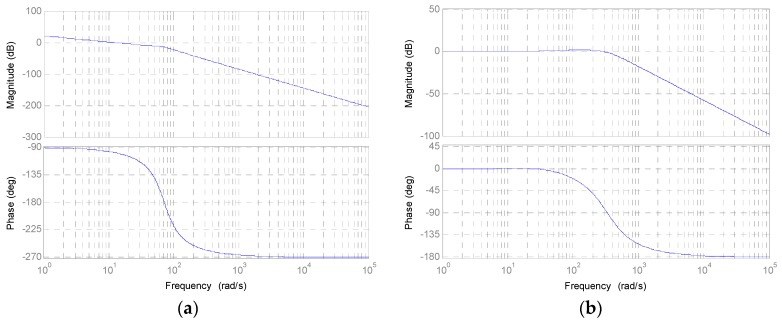
(**a**) Open loop frequency response of the direct drive actuator; (**b**) Closed loop frequency response of the direct drive actuator.

The transfer function in open loop frequency response is shown in Figure 15a and the closed loop frequency response with controllers in Figure 15b.

### 4.3. Dynamic Behavior Measurement and Direct Drive Model

**Figure 16:**
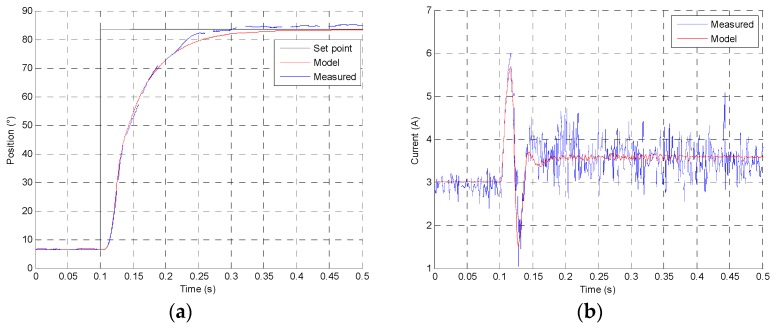
(**a**) Position step response comparison between measure and direct drive model; (**b**) Current response comparison between measure and direct drive model at 12 V and 25 °C conditions.

All components are defined according to the previous measurements. The model can be simulated at different set points and different temperatures, supply voltage and charge conditions. For a 77° increasing step, Figure 16a presents the response time at 12 V and 25 °C conditions with 170 mN·m charge, and in Figure 16b the current consumption comparison between the measure and the model is shown.

**Figure 17:**
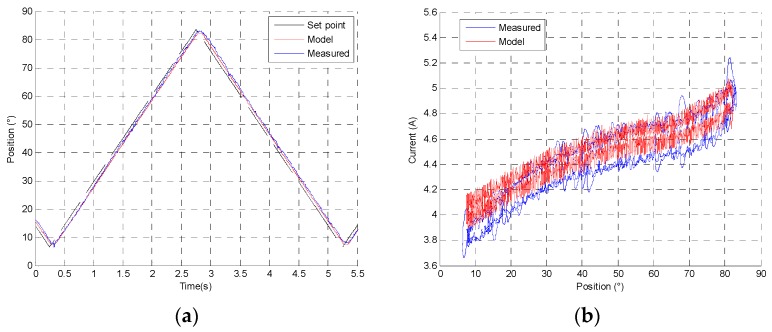
(**a**) Increasing and decreasing slope response comparison between measure and direct drive numerical model; (**b**) Current response comparison at 12 V and 25 °C conditions.

Figure 17a presents the position response of actuator for an increasing and decreasing 77° angular slope at 12 V and 25 °C conditions with 170 mN·m charge. Figure 17b shows the current machine evolution depends of friction and magnetic hysteresis according to the actuator position. A low resistance friction hysteresis can be observed, contrary to an indirect drive actuator.

### 4.4. Comparative Results between the Optimization and the Prototype

As shown in Table 3, the analytical method offers some sufficient results with a low deviation (<10%) between the numerical model and FEM results and then between the simulation results and the prototype measures.

The difference of rotor inertia values between the prototype and the analytical model is the additional parts to maintain the magnets on the rotor and to connect the actuator on the EGR system.

## 5. Actuator Energy Consumption with the EGR Load and Position Cycle

The direct drive actuator numerical model is coupled with the EGR system numerical model. Then the complete model is built in order to compute the energy consumption for all conditions of temperature and supply voltage.

### 5.1. Mechanical EGR System and Actuator Coupling

**Figure 18:**
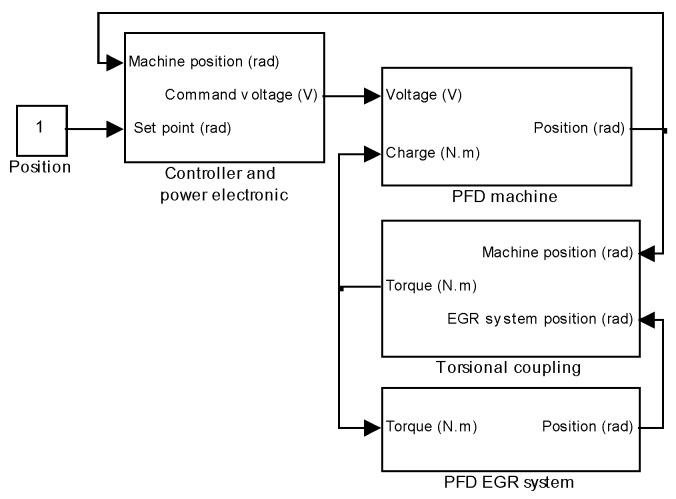
Actuator and EGR system coupling.

The actuator and valve system have been described by their electromechanical and dynamic equations. Figure 18 shows a schematic diagram combining the two numerical models. A torsional coupling is added between the actuator and EGR system as described in [17].

**Figure 19:**
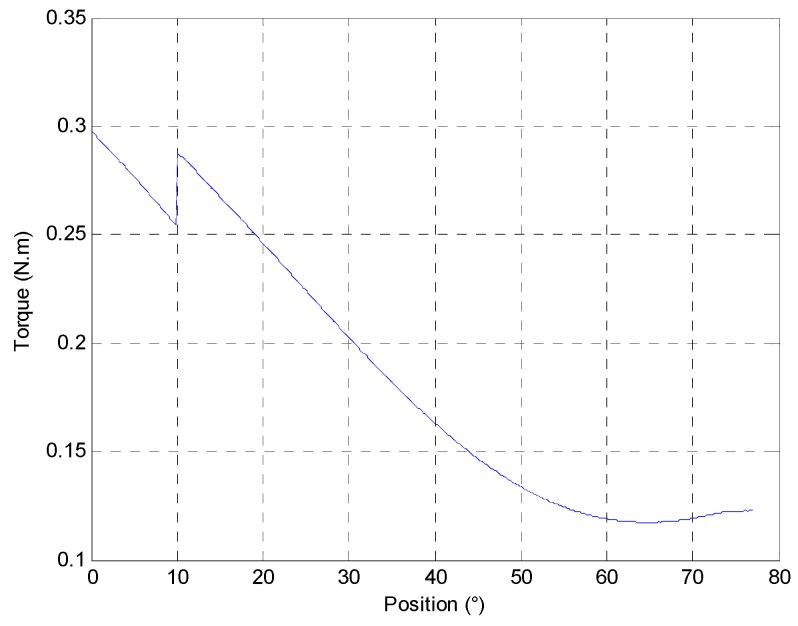
Load evolution according to the valve and flap position.

The resistive torque applied on actuator depends on the aerolic system (valve and flap) and the functioning condition of the ICE. The system has a 77° stroke. The worst case is defined by a resistive decreasing torque, as shown in Figure 19.

### 5.2. Increasing and Decreasing Position Step Response

**Figure 20:**
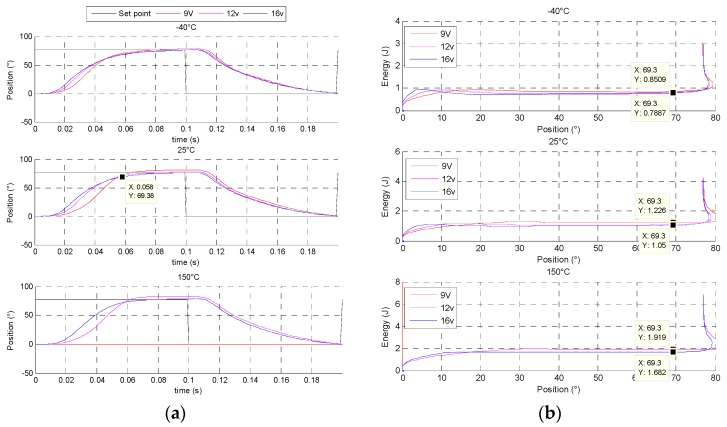
(**a**) Increasing and decreasing position step response of direct drive actuator at 25 °C; (**b**) Electrical energy consumption in increasing position step of direct drive actuator at 25 °C.

At 12 V and 130 °C, this machine reaches the objective with a 100 ms time response and a satisfactory time response quality. Figure 20a presents the mechanical behaviour in step response and Figure 20b shows the electrical energy for an increasing step. In transient behaviour, the direct drive actuator reaches 69.3° in 58 ms. It consumes 25% of electrical energy to move 90% of stroke and 75% of energy to do the remaining stroke and to keep the position.

The rotor inertia is minimized according to the optimized actuator geometry. This is a major advantage for reducing transient energy consumption. However to maintain the position, the major part of electrical energy is consumed in Joule losses.

### 5.3. Energy Consumption in 60 s Position EGR Cycle

**Figure 21:**
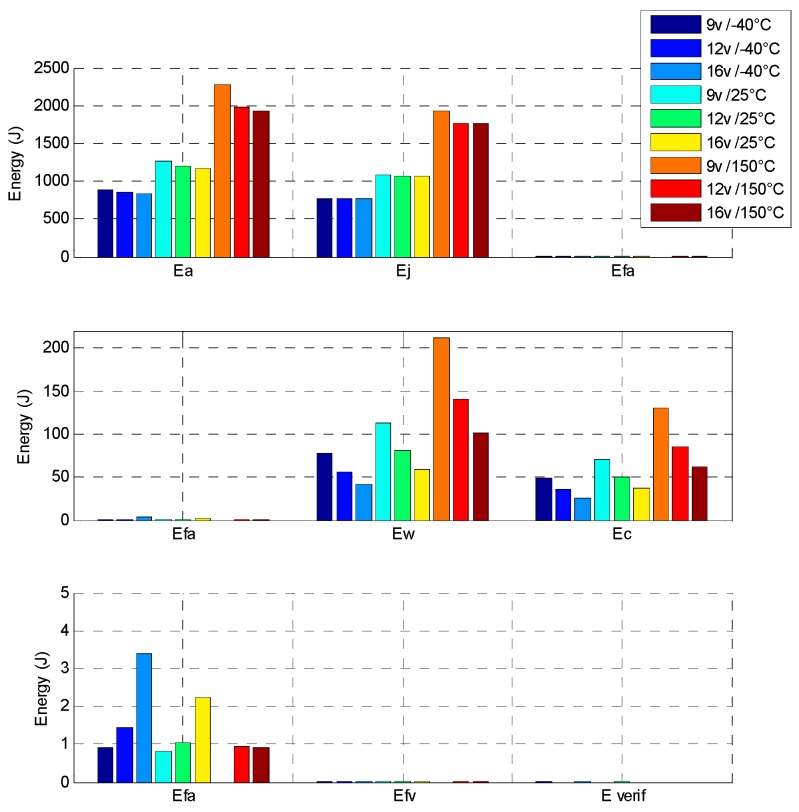
Direct drive energy balance at different voltages and temperatures. E_a_: the electrical energy consumption; E_j_: the Joule effect losses; E_fa_: the actuator mechanical losses; E_w_: the wire joule losses; E_c_: the H-bridge Joule losses and E_fv_: the valve mechanical losses.

Figure 21 displays the energy balance of the direct drive with a 60 s position EGR cycle, at different voltage and temperature conditions. All details about the energy balance are given. At 12 V and 25 °C, the direct drive consume a 1300 J electrical energy to realize the 60 s position EGR cycle. Some observations can be explained:

- when the temperature is increased, torque machine decreases according to the remanent flux density of magnets. On the contrary, the electrical resistance of wires increases, so for the same mechanical load level, the system consumes more electrical energy with high temperature level.
- the most of the energy consumption (90%) is dissipated in Joule losses inside the DC machine. The last 10% are mechanical losses inside gears and/or other Joule losses in resistive wire contact and commutation losses in H-bridge.

**Figure 22:**
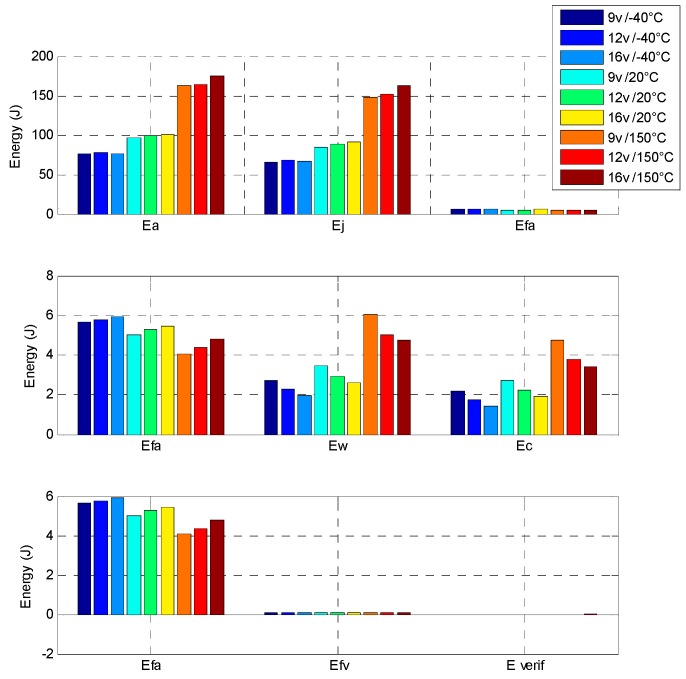
Indirect drive energy balance at different voltages and temperatures. E_a_: the electrical energy consumption; E_j_: the Joule effect losses; E_fa_: the actuator mechanical losses; E_w_: the wire joule losses; E_c_: the H-bridge Joule losses and E_fv_: the valve mechanical losses.

In order to compare these results, an indirect drive actuator, composed of a brushed DC machine with a spur gears set, is designed and optimized with the same requirements. The indirect drive actuator is simulated with the same conditions as the direct drive described in [7]. This actuator reaches the objective with a 150 ms time response and it cannot go faster. The mechanical behaviour in increasing step response shows that the indirect drive needs 65% of total electrical energy to move 90% of the stroke and 35% of energy for the remaining stroke. The reason for this is that the rotor inertia and the cogging torque are major disadvantages for brushed DC machines, as it leads one to design a strong failsafe spring and as a consequence to increase the transient energy consumption against the spring. Figure 22 shows the energy balance of the indirect drive with the 60 s position EGR cycle, at the same different voltage and temperature conditions.

The indirect drive actuator need only 100 J electrical energy to realize the 60 s position EGR cycle. The EGR position cycle is dynamically simple and has few requirements to maintain a stable position for a long time.

## 6. Conclusions

To conclude the study of this prototype, the method of calculation has been proven successful by comparison between the analytical model and the prototype. By the way, it has been demonstrated that the direct drive actuator reached 69.3° in 58 ms in transient behaviour while it consumes 25% of the electrical energy to move 90% of the stroke and 75% of energy to do the remaining stroke and to keep the position. On the other hand, the indirect drive actuator, reaching 69.3° in 120 ms, consumes 65% of the total electrical energy to move 90% of the stroke and 35% of the energy for the remaining stroke. Moreover, in this application, the indirect drive actuator needs only 100 J electrical energy to realize the 60 s position EGR cycle compared to the direct drive which consumes a 1300 J electrical energy. Consequently, in this given case, the EGR position cycle has shown that most part of the cycle has to keep the position and the indirect actuator is better suited for this action. Also, it should be noted that the direct drive would be needed when the applications are faster. This is interesting for ultra-fast and constantly moving applications with a low torque in order to minimize the electrical consumption and for its higher level of reliability. Given the choice of the position cycle of an application, a decision must be made between the choice of a direct or an indirect actuator. It is then important to emphasize the choice of this position cycle.

## Figures and Tables

**Table 1:** Optimized parameter values.

Parameters		Unit	Min. Limit	Initial Value	Max. Limit	Optimal Value
Magnet thickness ratio	h_a_	-	0.001	2/62	0.9	0.043
Pole height ratio	h_n_	-	0.1	21.5/31	0.9	0.7271
Polar teeth opening angle	aon	°	100	124	160	117
Coil thickness	E_b_	mm	2	5.4	8	5.74
Copper diameter	d_cu_	mm	0.1	0.9	2.5	0.9
External raduis	R_ext_	mm	10	35	30	32.6
External height	H_ext_	mm	10	62	51	59.4
Forward accelerating time	T_a_	ms	1	87	149	86.8
Backward accelerating time	T_r_	ms	1	5.4	149	5.3

**Table 2:** Linear magnetics results of optimized actuator.

Results	Performance	Unit	Analytic	FEM	Deviation %
Magnet flux density	B_ea_	mT	597	590	1.1
Fmm flux density	B_ni_	mT	278	262	6.1
Air gap flux density	B_e_	mT	875	867	<1
Saturation magnetic flux at 130°	Φ_sat_	µWb	627	587	7
Actuator torque at saturation current	T_m_	N.m	0.497	0.489	1.6
Magnetomotive force	Ni	A.t	445.95	445.32	<1

**Table 3:** Performance comparison.

Result at 25 °C		Unit	Analytical	FEM	Prototype
Electrical resistance	R	Ω	1.12	N C	1.2
Electrical Inductance	L	H	24	22	21
Torque constant	K_t_	mN·m/A	90	89	84
Inertia rotor	J_m_	kg·m^2^	0.8 × 10^−5^	N.C	1.96 × 10^−5^
